# Supplementary material for: CDK7, CDK9, or CDK11 inhibition reduces neutrophil-driven inflammation and tissue damage in experimental autoimmune models
Source: Cell Commun Signal. 2026 Sep 30;24:520. doi: 10.1186/s12964-026-03219-x (PMC13628873; doi:10.1186/s12964-026-03219-x)
Supplement: Supplementary file 1 [file 12964_2026_3219_MOESM5_ESM.docx]

**Fig. S1: IC-stimulation of human neutrophils induces changes in gene expression without affecting viability or apoptosis.** Purified human neutrophils were stimulated with immobilized hCOL7 anti-COL7 IgG1-IC for 6 h at 37 °C and 5 % CO_2_. **(A)** Lysates were prepared for bulk RNAseq and analyzed by DESeq2. (B) Differentially expressed genes among selected CDK regulators were determined and log2 fold changes (log2 FC) are displayed for the individual samples. Significantly changed genes (p_adj_ < 0.1) are highlighted in grey. **(C)** Cells were stained for activation markers and Annexin V/Zombie NIR to assess viability and apoptosis after stimulation by flow cytometry. Mann Whitney test was performed. ns, not significant. **(A, B)** n = 3. **(C)** n = 5.

**Fig. S2: CDK1i Ro 3306 and CDK7i THZ1 2HCl reduce CD62L shedding in IC-stimulated human PMNs.** **(A-K)** Human PMNs were stimulated for 2 h with hCOL7 anti-COL7 IgG1-IC and subsequently analyzed for CD62L shedding. Inhibitors targeting specific CDKs were tested in flow cytometry for their ability to minimize CD62L shedding. Based on FSC-SSC gating, alive singlet CD45^+^ cells were considered. The results were normalized to the positive control stimulated with IgG1-IC but without inhibitor indicated by the red line. The dose-response curves were calculated and the IC_50_/EC_50_ values are indicated if in the tested concentration range. Cells only, antigen only (Ag), and antibody only (Ab) served as negative controls. Kruskal-Wallis test with Dunn’s multiple comparisons test was performed. * p ≤ 0.05. ** p ≤ 0.01. *** p ≤ 0.001. n = 5.

**Fig. S3: CDK1i Ro 3306 or CDK7i THZ1 2HCl decrease CD18 expression on IC-activated human PMNs.** **(A-K)** Following stimulation of human PMNs for 2 h with hCOL7 anti-COL7 IgG1-IC and treatment with CDKi, the cells were analyzed for their CD18 expression in flow cytometry. Based on FSC-SSC gating. alive singlet CD45^+^ cells were considered. The results were normalized to the positive control stimulated with IgG1-IC but without inhibitor indicated by the red line. The dose-response curves were calculated and the IC_50_ values are indicated if in the tested concentration range. Cells only, antigen only (Ag), and antibody only (Ab) served as negative controls. Kruskal-Wallis test with Dunn’s multiple comparisons test was performed. * p ≤ 0.05. ** p ≤ 0.01. n = 5.

**Fig. S4: Adhesion of IC-activated human PMNs is not significantly reduced by the additional CDKi used.** **(A-K)** Human PMNs were stimulated with hCOL7 anti-COL7 IgG1-IC for 2 h. Impedance-based measurement of adhesion was conducted using inhibitors targeting specific CDKs. The area under the curve was determined and the results were normalized to the positive control stimulated with IgG1-IC but without inhibitor indicated by the red line. The dose-response curves were calculated and the IC_50_/EC_50_ values are indicated if in the tested concentration range. Cells only, antigen only (Ag), and antibody only (Ab) served as negative controls. Kruskal-Wallis test with Dunn’s multiple comparisons test was performed. * p ≤ 0.05. ** p ≤ 0.01. *** p ≤ 0.001. n = 5.

**Fig. S5: Inhibition of CDK1, CDK2, CDK4/6, CDK7, or CDK9 in IC-stimulated human PMNs minimizes ROS release.** **(A-K)** Activation of human PMNs with hCOL7 anti-COL7 IgG1-IC for 2 h, treatment with selective CDKi and subsequent chemiluminescence-based analysis of ROS release was performed. The area under the curve (AUC) was calculated and the results were normalized to the positive control stimulated with IgG1-IC but without inhibitor indicated by the red line. The dose-response curves were calculated and the IC_50_ values are indicated if in the tested concentration range. Cells only, antigen only (Ag), and antibody only (Ab) served as negative controls. Kruskal-Wallis test with Dunn’s multiple comparisons test was performed. * p ≤ 0.05. ** p ≤ 0.01. *** p ≤ 0.001. n = 5.

**Fig. S6: Apoptosis in IC-stimulated human PMNs is increased by application of CDK1/2i, CDK12i, or CDK8/19i.** **(A-K)** Human PMNs were activated with hCOL7 anti-COL7 IgG1-IC for 2 h. After using selective CDKi, flow cytometry was performed. Based on FSC-SSC gating, singlet CD45^+^ CD16^+^CD14^-^CD193^-^ cells were analyzed. Annexin V^+^ Zombie NIR^-­ ­­^cells were considered apoptotic. The results were normalized to the positive control stimulated with IgG1-IC but without inhibitor indicated by the red line. The dose-response curves were calculated and the EC_50_ values are indicated if in the tested concentration range. Cells only, antigen only (Ag), and antibody only (Ab) served as negative controls. Kruskal-Wallis test with Dunn’s multiple comparisons test was performed. * p ≤ 0.05. ** p ≤ 0.01. *** p ≤ 0.001. For apoptosis experiment using Palbociclib HCl, AS2863619, BMS-265246, and SR-4835 n = 6, rest n = 5.

**Fig. S7: Total percentage of apoptotic cells is comparable across inhibitor-treated and control cells. (A-N)** Human PMNs were treated with hCOL7 anti-COL7 IgG1-IC for 2 h. After application of selective CDKi, flow cytometry was performed. Based on FSC-SSC gating, singlet CD45^+^ CD16^+^CD14^-^CD193^-^ cells were analyzed. Annexin V^+^ Zombie NIR^-­ ­­^cells were considered apoptotic. The total percentage of apoptotic cells among the CD45^+^ cells is displayed. Kruskal-Wallis test with Dunn’s multiple comparisons test was performed. Cells only, antigen only (Ag), and antibody only (Ab) served as negative controls. Cells treated with immune complexes only (IC) served as positive controls. For apoptosis experiment using Palbociclib HCl, AS2863619, BMS-265246, and SR-4835 n = 6, rest n = 5.

**Fig. S8: Viability of IC-treated human PMNs is minorly affected by CDK1, CDK7, and CDK12 inhibition.** **(A-K)** Human PMNs stimulated with hCOL7 anti-COL7 IgG1-IC and treated with CDKi for 2 hr were analyzed for toxic effects in flow cytometry. Based on FSC-SSC gating, singlet CD45^+^ cells were studied. Annexin V^-^ Zombie NIR^-^ cells were considered viable. The results were normalized to the positive control stimulated with IgG1-IC but without inhibitor indicated by the red line. The dose-response curves were calculated and the IC_50_ values are indicated if in the tested concentration range. Cells only, antigen only (Ag), and antibody only (Ab) served as negative controls. Kruskal-Wallis test with Dunn’s multiple comparisons test was performed. * p ≤ 0.05. n = 5.

**Fig. S9: CDK1i Ro 3306 or CDK4/6i palbociclib HCl do not reduce the AESA in local EBA**. **(A-B)** EBA was induced by injection of anti-mCOL7^c^ IgG and the mice were treated systemically with three more CDKi. The affected ear surface area (AESA) was evaluated. Mixed-effects analysis with Šidák’s multiple comparisons test was performed. *** p ≤ 0.001. **(A)** n = 7, **(B)** n = 4.
