# Supplementary material for: CDK7, CDK9, or CDK11 inhibition reduces neutrophil-driven inflammation and tissue damage in experimental autoimmune models
Source: Cell Commun Signal. 2026 Sep 30;24:520. doi: 10.1186/s12964-026-03219-x (PMC13628873; doi:10.1186/s12964-026-03219-x)
Supplement: Supplementary file 2 [file 12964_2026_3219_MOESM6_ESM.docx]

**Supplemental table 1.** Additional targets of CDKi used adopted from Selleckchem.com and references for *in vivo* application and current stage of drug development.

| Substance | Main target | Additional targets (IC_50_/*Kd*/Ki) | Reference | Stage of drug development |
| --- | --- | --- | --- | --- |
| Ro 3306 | CDK1 | PKCδ (318 nM), SGK (497 nM), ERK (1980 nM) | [114] | preclinical |
| OTS964 | CDK11B | TOPK (28 nM) | [115] | preclinical |
| Palbociclib HCl | CDK4/6 | CDK4-cyclin D1 (11 nM), CDK6-cyclin D2 (15 nM) | [116, 117] | FDA- and EMA-approved |
| THZ1 2HCl | CDK7 | - | [118, 119] | preclinical |
| THZ2 | CDK7 | - | [100] | preclinical |
| MC180295 | CDK9 | CDK4-cyclin D (112 nM), CDK1-cyclin B (138 nM), CDK5-P35 (159 nM), CDK5-P25 (186 nM), CDK2-cyclin A (233 nM), CDK2-cyclin E (367 nM), CDK3-cyclin E (399 nM) | [82] | preclinical |
| LY3143921 hydrate | CDC7 | - | [120] | clinical trial |
| BMS-265246 | CDK1/2 | CDK2-cyclin E (9 nM), CDK4-cyclin D (230 nM) | [121] | preclinical |
| Purvalanol B | CDK1/2/5 | CDK2-cyclin A (6 nM), CDK5-P35 (6 nM), CDK2-cyclin E (9 nM) | [122] | preclinical |
| BSJ-4-116 | CDK12 | (CDK12 degrader) | [123] | clinical trial |
| SR-4835 | CDK12/13 | CDK12 (99 nM, *98 nM*) | [124] | preclinical |
| K03861 | CDK2 | CDK2 (A144C, 15.4 nM), CDK2 (C118L, 18.6 nM), CDK2 (WT, 50 nM) | [125] | preclinical |
| AS2863619 | CDK8/19 | CDK19 (4.277 nM) | [126] | preclinical |
| BAY1251152 | CDK9 | - | [127] | clinical trial |

**Supplemental table 2.** Descriptive statistics for all cytokines analysed in KBxN STA at day 0 and day 7.

|  | day 0 | | | day 7 | | |
| --- | --- | --- | --- | --- | --- | --- |
|  | **median ± SEM [pg/mL]** | | **p value** | **median ± SEM [pg/mL]** | | **p value** |
| cyto-kine | **treated** | **control** | **treated vs. control** | **treated** | **control** | **treated vs. control** |
| GM-CSF | 0.564 ± 0.3987 | 0 ± 0 | 0.4444 | 1.036 ± 0.6401 | 1.465 ± 0.7801 | 0.5556 |
| IL-10 | 13.99 ± 8.837 | 16.63 ± 16.63 | 0.6825 | 27.06 ± 9.973 | 60.15 ± 9.976 | 0.0317 |
| IL-12p70 | 1.58 ± 0.8095 | 0.69 ± 0.3984 | 0.5714 | 1.546 ± 1.029 | 3.53 ± 1.436 | 0.3095 |
| IL-17A | 1.144 ± 0.5896 | 0.44 ±  0.2 | 0.5556 | 1.53 ± 0.4803 | 1.47 ± 0.3853 | 0.9048 |
| IL-1α | 190 ±  41.09 | 218.5 ± 33.07 | 0.5556 | 15.69 ± 4.246 | 14.89 ± 5.507 | 0.7302 |
| IL-1β | 7.224 ± 4.069 | 4.81 ± 1.707 | > 0.9999 | 3.076 ± 1.342 | 7.043 ± 1.508 | 0.1032 |
| IL-23 | 29.37 ± 11.81 | 15.08 ± 9.157 | 0.4365 | 43.63 ± 17.1 | 25.55 ± 6.944 | 0.5476 |
| IL-27 | 124.5 ± 32.39 | 133.7 ± 85.19 | 0.7302 | 116.6 ± 41.59 | 111.8 ± 18.37 | 0.375 |
| IL-6 | 4.162 ± 1.272 | 2.088 ± 1.033 | 0.1746 | 2.824 ± 0.8739 | 34.84 ± 5.281 | 0.0159 |
| IFN-β | 78.04 ± 20.83 | 37.03 ± 27.01 | 0.4127 | 10.04 ± 6.495 | 29.55 ± 7.633 | 0.0952 |
| IFN-γ | 3.578 ± 1.287 | 2.833 ± 0.9399 | 0.7937 | 2.968 ± 0.6265 | 5.545 ± 0.9739 | 0.127 |
| MCP-1 | 12.09 ± 2.878 | 7.603 ± 3.671 | 0.2857 | 9.492 ± 5.536 | 16.65 ± 4.481 | 0.3175 |
| TNF-α | 13.24 ± 2.719 | 11.38 ± 1.989 | 0.4444 | 10.42 ± 3.304 | 22.59 ± 6.41 | 0.0952 |
